# SAMGRID: Security Authorization and Monitoring Module Based on SealedGRID Platform †

**DOI:** 10.3390/s22176527

**Published:** 2022-08-30

**Authors:** George Suciu, Aristeidis Farao, Giorgio Bernardinetti, Ivan Palamà, Mari-Anais Sachian, Alexandru Vulpe, Marius-Constantin Vochin, Pavel Muresan, Michail Bampatsikos, Antonio Muñoz, Christos Xenakis

**Affiliations:** 1 R&D Department, Beia Consult International, 41386 Bucharest, Romania; 2 Department of Digital Systems, University of Piraeus, 18534 Piraeus, Greece; 3 Dipartimento di Ingegneria Elettronica, University of Rome Tor Vergata, 00133 Rome, Italy; 4 Telecommunications Department, University POLITEHNICA of Bucharest, 61071 Bucharest, Romania; 5 Department of Computer Science, University of Malaga, 29016 Malaga, Spain

**Keywords:** smart energy, smart grid, IoT, FI-WARE, policy, security

## Abstract

IoT devices present an ever-growing domain with multiple applicability. This technology has favored and still favors many areas by creating critical infrastructures that are as profitable as possible. This paper presents a hierarchical architecture composed of different licensing entities that manage access to different resources within a network infrastructure. They are conducted on the basis of well-drawn policy rules. At the same time, the security side of these resources is also placed through a context awareness module. Together with this technology, IoT is used and Blockchain is enabled (for network consolidation, as well as the transparency with which to monitor the platform). The ultimate goal is to implement a secure and scalable security platform for the Smart Grid. The paper presents the work undertaken in the SealedGRID project and the steps taken for implementing security policies specifically tailored to the Smart Grid, based on advanced concepts such as Opinion Dynamics and Smart Grid-related Attribute-based Access Control.

## 1. Introduction

Traditional power grid models are based on a central system for generating and distributing energy and have undergone significant changes in recent years [1]. The integration of the latest generation of technologies, rare in critical infrastructure such as the Internet of Things (IoT), has facilitated the evolution to a more dynamic and connected power grid model now known as the Smart Grid (SG). SG’s contributions result from introducing a mutual flow of information between manufacturers and customers, from which both can benefit. This flow enables fine-grained consumption measurements reported to each energy service provider in near real-time to provide consumers with up-to-date price data or control a utility that contains the grid’s energy load in real-time according to actual demand, allowing utilities to perform accurate demand response procedures by anticipating high demand peaks, avoiding and mitigating power outages, and distributing the load on available generators. On the other hand, consumers can take part in programs that reduce electricity consumption in the event of rising energy prices while using home-generated (renewable) electricity (such as the so-called microgrid).

The above measurement model is called Advanced Metering Infrastructure (AMI) [2]. Technically speaking, this infrastructure consists of several interconnected elements that collect home-measured consumption data, later passed to the power company via an aggregation point. Part of this information is analyzed through Meter Data Management Systems [3]. As a result, further control procedures for the system include industry and information technology equipment (integrated throughout the infrastructure) and correct usage of devices and resources by all involved parties. The architecture for capturing measurement information from IoT devices and consistently controlling power generation contributes to the development of cybersecurity attacks that can compromise resource availability and thus network stability. Access control is essential to manage permissions for all users, processes, and heterogeneous devices that interact continuously within the infrastructure in this complex environment. Therefore, it is imperative to consider the full range of requirements for this scenario to apply the available solutions accurately and propose hybrid access control mechanisms with integrated security monitoring mechanisms. 

This paper uses the approval component to address all these requirements in a modular and flexible way while defining fine-grained guidelines for monitoring the security status of each participating node being an integrated part of a hierarchical authentication framework that spans different devices. The approval framework adheres to integrated industry standards and robust policy rules that always consider the state of the context. In this case, the context awareness manager is used, an authorization component that uses the authentication module in the scope of validating the element’s identity requesting access to the resource and signing the token received from the appropriate authorization unit. These components can be put together to make timely access control decisions without impacting the throughput of network assets or ensuring the lowest level of security at all times.

The joining of all these components can certainly help issuing access control decisions in a timely manner without interfering in the throughput of the network assets and ensuring a minimum level of security at all times, as it is envisioned in Table 1 below.

The paper makes the following additional contributions in respect to the already obtained project results:

- providing security and privacy requirements for a module dedicated to delivering security authorization and monitoring the security status of the participating nodes;
- proposing SAMGRID, a novel authorization and security monitoring module tailored to SG needs based on well-established security technologies;
- assessing SAMGRID’s performance: implementation and evaluation were performed in a simulation environment.

This work is carried out as follows: Section 2 presents essential basic information on authorization in the SG, the motivation of our work and the security and functional requirements that we have defined based on the needs of stakeholders. Section 3 follows, where the related works are discussed, while Section 4 describes the SAMGRID concept. Furthermore, Section 5 contains an assessment of the measurable performance of SAMGRID, and Section 6 examines its security properties. Finally, Section 7 concludes the paper.

## 2. Authorization in SG

### 2.1. Definition and Participants

In this paper, we focus on the importance of authorizing and monitoring the security status of the participating entities within a SG network. The main SG components are the Utility, the Smart Meter (SM) and the Aggregator. The Utility is responsible for billing by computing the total consumption of a customer at the end of a billing period. The SM is placed within a house or building, and its purpose is to collect the readings of the electricity consumption. The Aggregator represents the binder between the Utility and the SMs. It is responsible to sum all the readings received by SMs and transmit the results to the Utility. In this way, data become available without putting too much load on the Utility. In general, SG’s main goal is to provide a dynamic two-way information exchange between Utility companies and their customers contributing towards a smart and sustainable energy management. However, in such cases, the main challenges that a SG has to exceed are related to scalability [14], trust [15] and interoperability [15]. Thus, divergent information and operational technologies have to cooperate for achieving interconnection of various mechanisms. 

**Figure 1:**
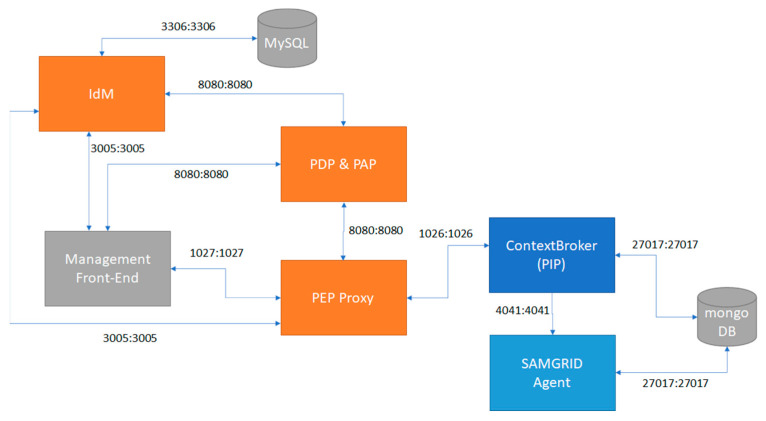
Illustration of participants in the authorization component of SAMGRID.

There are many ways to cope with the aforementioned challenges including but not limited to policy-based management. Thus, policy specification languages are utilized to communicate the various authorization policies in numerous access control applications with complicated policies. A well-known as well as commonly used language in SG ecosystems is the XACML [16], which is used to construct complex authorization policies [17]. The entities that participate under the policy manner are the following: (i) Policy Enforcement Point (PEP): this is responsible for performing the decision requests, receives policy updates and accordingly translates them, as well as enforces the decisions that stem from each policy. (ii) Policy Decision Point (PDP): this assesses the applicable policy against other relevant policies and attributes providing the decision outcome to PEP. (iii) Policy Information Point (PIP): this acts as a source of attribute values to make a policy decision. (iv) Policy Administration Point (PAP): this provides the authoring and the maintenance of a policy or a set of policies. As we can observe, in the SG ecosystem the participating entities own numerous titles. For instance, a Utility in a domain may also be a PDP. That leads to the fact that the different roles can be allocated to an entity being hardcoded in a device. Figure 1 shows the involved entities and the flows between them.

### 2.2. Motivating Examples

In this section, we will showcase the most notorious cyber-attacks in critical infrastructure that occurred in recent decades to gain a better understanding of the presented notions. Our approach aims to emphasize not only the security flaws that enable cyberattacks in critical infrastructures, but also how a malicious actor can take advantage of various vulnerabilities and launch attacks. Moreover, the following examples come from real-life events that shocked involved governments, citizens and stakeholders, also these are explained in brief providing valuable insights.

**Stuxnet** was a directed cyberwarfare attack against the Iranian nuclear program. It was first uncovered in 2010; however, it has been reported that it was in development since at least 2005. The attackers’ approach relied on delivering the worm via USB sticks and local networks. Stuxnet infected both Windows PCs and also controllers. However, its behavior against the controllers was totally different, picking controllers from a specific manufacturer. Once Stuxnet identified its targeted controller then it went through an intricate process of fingerprinting to make sure that it was the target. When it met the requirements, Stuxnet’s dropper loaded rogue code to the controller. The code injection enables Stuxnet to stealthily launch its code, letting legitimate code continue correctly working. The rogue code periodically worked. When the attack time came, the rogue code took control without letting the legitimate controller code understand. Finally, during the attack, the genuine code of the controller was knocked out [18].

Another infamous example is **BlackEnergy**, which is the first reported successful cyberattack on a power grid. On 23 December 2015, the attack occurred, managing to disrupt three energy distribution companies in Ukraine and temporarily stop the electricity supply to the end users. In particular, the attacking group that mounted the attack utilized spear-phishing emails attaching malicious Excel documents with macros infecting computers in a targeted network. Additionally, it obtained the credentials and hijacked the Supervisory Control and Data Acquisition (SCADA) systems to ultimately switch off certain substations. At the same time, the attackers flood the call centers with automated telephone calls, preventing the affected utilities from receiving outage reports from their customers (end-users) confronting the response effort [19,20].

Additionally, a well-known attack is **GreyEnergy** targeting critical infrastructure organizations in Central and East Europe in 2018. It is widely known that the malware used during this attack bears many similarities to the one used in the BlackEnergy attack (see above). The attacking group that was responsible for this cyber-attack used two ways to achieve the intrusion into the organization’s network. One the one hand, the first weapon they used was through the *GreyEnergy mini*, which is a first-stage backdoor that works without the demand of administrative privileges- the attackers searched for public-facing web services running on servers that were connected on the targeted network. Once it was finished, the attackers started mapping and scanning the network, as well as collecting credentials to obtain administrator privilege. Then, they were capable of initiating the main malware. In particular, the attackers targeted servers with high uptime, and workstations used to control industrial and control system environments. Additionally, they utilized command and control services to establish communication among their computers (malicious network) and the compromised machines (targeted network). On the other hand, the second way to end the targeted network was via spear phishing emails that bear with them malicious attachments.

The cyberattacks in industrial control systems (ICS) are not a cybersecurity issue that belongs to the past, in 2020 a ransomware encrypted data in **Düsseldorf Hospital** and then demanded ransom to unlock it. During this attack, the first death by ransomware was reported. Particularly, the ransomware compromised the digital infrastructure that the hospital relies on to organize its processes forcing the cancellation of many operations and other procedures. The ransomware entered the University Hospital Düsseldorf’s network through a widely known vulnerability in a Citrix application [21]. Apart from this attack, in 2021 **Colonial Pipeline**—the largest fuel pipeline in the U.S.A.—shut down for five days due to a ransomware attack [22]. In this case, the attackers managed to compromise the targeted network utilizing a VPN account. They found the related credentials inside a batch of leaked passwords on the dark web [23,24].

Apart from the aforementioned attacks, it is also mandatory to analyze the different attack stages to complete a cyberattack in ICS. At this point, we will mention the most well-known frameworks that provide the necessary steps for an attack. On the one hand, there is the Cyber Kill Chain framework [25,26] that provides seven steps that attackers have to fulfill to achieve their objectives, the steps are the following: (i) Reconnaissance; (ii) Weaponize; (iii) Delivery; (iv) Exploitation; (v) Installation; (vi) Command and Control and (vii) Actions on objectives. On the other hand, there is the MITRE ATT&CK framework [26,27] that provides 14 steps, which the attackers have to follow to accomplish their attack. The steps for this are the following: (i) Reconnaissance; (ii) Resource Development; (iii) Initial Access; (iv) Execution; (v) Persistence; (vi) Privilege Escalation; (vii) Defense Evasion; (viii) Credential Access; (ix) Discovery; (x) Lateral Movement; (xi) Collection; (xii) Command and Control; (xiii) Exfiltration and (xiv) Impact. Both frameworks follow the same pattern. The primary difference between the aforementioned frameworks is that the MITRE ATT&CK framework is a list that consists of tactics and techniques; we have to note that it does not propose a specific order of operation. However, the Cyber kill Chain proposes a well-defined sequence of events.

### 2.3. Security and Functional Requirements

As we can observe, SG is an ecosystem that inherits risks that are directly related not only to the participating SG components (Smart Meter, Aggregator, Utilities), but also to the inadequate security controls implemented by handlers to these. This leads to the conclusion that security and functional requirements need to be declared for a scheme that aims to provide authorization and security monitoring features. Since the functional and security requirements of a SG ecosystem have been extensively expressed by the literature, we aim to shed light on requirements related to security and functionality being dedicated to authorization. In particular, we formulate the requirements intending to meet high demands of SG stakeholders. At the end, we have to note that we express the ensuing requirements adopting a security by design approach. 

#### 2.3.1. Security Requirements

Since the security among a SG ecosystem depends not only on the devices (e.g., vulnerabilities), but also on the poor security practices that are established for authentication and authorization purposes, we express a kit of standard security requirements applied to it [16,28,29,30,31,32].

S1. Data confidentiality: Data exchanged within a SG ecosystem should be available only to SG components with the respective privilege.
S2. Data integrity and authenticity: Data exchanged among the participating SG components should be safeguarded against alteration and replication; thus, these should be capable of verifying the origin of the acquired data.
S3. Accountability: Devices, handlers/employees and end-users should be accountable for their actions.
S4. Non-repudiation: Devices, handlers and end-users should not be able to deny their actions.
S5. Physical protection: All electronic devices that participate in a SG ecosystem should contain protection mechanisms to prevent being tampered by adversaries with physical access.

#### 2.3.2. Functional Requirements

Apart from the security requirements, a SG ecosystem consists of processes that demand specific functionalities to be enabled. Analyzing the current literature, we express the functional requirements applying a security by design approach but understanding the stakeholders demands [28,29,30,31,32].

F1. Time consuming: As it is well known, the SG concept aims to support real-time services to its end-user. Thus, the implemented application for authentication, authorization, policy updating should not consume much time and deplete the available sources.
F2. Scalability: A SG ecosystem should consist of applications that are capable of handling the numerous fluctuations of grid’s size (e.g., nodes can join and leave a grid) without negatively affecting their performance.
F3. Delegated access control: Any application access must be authenticated and authorized by a security policy, and the granting decisions must be made relying on a trusted party.
F4. Authorization: Any access to applications must be authorized according to a security policy.
F5. Authentication: Requesters should be authenticated before accessing any application.

## 3. Related Work

The literature in the field of SAMGRID contains the security authorization and opinion dynamics approaches that have been designed for SG ecosystems and the techniques that are used for monitoring the security status of the participating nodes.

### 3.1. Security Authorization Approaches

Although the literature proposes different authorization methods and mechanisms for the SG ecosystem, to the best of our knowledge, this is the first paper that proposes the seamless work of an opinion dynamics approach together with an individual Authorization mechanism. This work is an extension to the paper “FI-WARE authorization in a Smart Grid scenario” written by George Suciu, Cristiana Istrate, Mari-Anais Sachian, Alexandru Vulpe, Marius Vochin, Aristeidis Farao and Christos Xenakis, which has been published in the proceedings of the 4th Global Internet of Things Summit (GioTS) in 2020. Some of the extensions of the work include: (i) an elaborated description of security and functional requirements that should be accomplished by a proposed solution for authorization purposes in SG ecosystem; (ii) a proposal of an opinion dynamics approach that works together with the initial version of the Authorization element proposed in [18]; (iii) a summary of several performance evaluation experiments performed to analyze different aspects of our module demonstrating the impact that the proposed solution has in terms of performance and efficiency and (iv) an analysis related to the security features of SAMGRID. Parts of the work presented in [18] are reused in the current paper.

Security interoperability known as one of the most challenging research areas within the field of critical infrastructures by International Organizations such as the NIST, and IEEE. Therefore, diverse technologies (sensors, meters, actuators, etc.) and various communication systems (WiMAX, Wi-Fi, ZigBee, 3G cellular, etc.) as well as different domains must cooperate in a unified ecosystem to provide the ability of performing critical actions. These actions, involving the control of user’s sensitive information (e.g., electrical consumption) performed across the different elements of the SG may be (i) tampered by malicious actors if data are not completely protected, or (ii) disrupted due to missing standardization and interoperability mechanisms. The design of secure authorization and interoperability mechanisms is a complex process as specified in [4,5]. It is stated that the interconnection between systems that were not originally envisioned to interoperate may pose unanticipated problems, not just in operation, but in data availability, resolution, and format; it may also cause significant delays in the primitive operations.

A solution for providing a decentralized SG in a secure manner using blockchain is presented in [6]. In respect to Electricity Theft Detection, in [7] two solutions based on supervised learning are proposed. The first solution addresses class imbalanced problems solving, perform feature extraction and then use a deep learning-based system to classify electricity consumers. The second solution is a Synthetic Minority Oversampling Technique Edited Nearest Neighbor (SMOTEENN) system. [8] improves the security of existing SCADA systems within smart grids using a cyber-physical digital signature scheme. In [9], Advanced Metering Infrastructure (AMI), which is an important component of an IoT based Smart Grid is analyzed separately and secured based on evolutionary game theory. Ref. [10] proposed a middleware architecture based on RBAC, Policy Enforcement Points (PEPs) and Policy DecisionPoints (PDPs) to collect data streams from several sources connected to the AMI in a standardized format. In [11], a solution based on the usage of PEPs and PDPs has been proposed to interconnect large distributions, involving technologies of different infrastructures, manufacturers and vendors. In [12], a data-centric access control framework for smart grids that follow the publish/subscribe model has been analyzed, adopting an Attribute-Based Authorization Policy. In [13], an authorization mechanism for monitoring and reduction in resource consumption by using resource trading contribution, implemented on blockchain technology, has been designed. The proposed system provides secure data access and storage together with controller functions transfers among householders.

The main limitation of the related state of the art is that these solutions are not able to cope with the dynamic environment of SG, since they are based mainly on RBAC. Even more, the above solutions do not offer any implementation details, nor performance evaluations through simulations.

Itron’s OpenWay Riva [33] is a commercial communication platform that offers well-defined points of interoperability between customer and utility systems, greatly simplifying and reducing integration costs and issues.

### 3.2. Opinion Dynamics Approaches

Smart Grid is one of the largest applications of the Internet of Things, the revolution of the Internet and machine-to-machine (M2M) communications. While SG offers many well-known benefits and new opportunities, their distributed nature and two-way information flow between consumer and producer enables a multitude of new attacks against smart grid infrastructure. Given the potentially extremely severe consequences that these attacks could have (e.g., environmental hazards/pollution, rendering hospitals or security defenses inoperable, suspension of economic activities) it is important to note that these attacks are likely to have a significant impact on the environment. Therefore, it is evident that it is imperative to develop anomaly/intrusion detection techniques and systems.

Traditional Intrusion Detection Systems (IDS) are only a first line of defense in attempting to identify anomalous behavior at very specific points in the infrastructure and are tailored to specific types of communication standards or data types, which is not sufficient to track the wide range of attack vectors that could be used against an SG environment. One of the most interesting and innovative cybersecurity innovations in the SG scenario is the usage of the Opinion Dynamics as a distributed detection technology to evaluate the security status of the SG environment. The Opinion Dynamics method proposes to aggregate the coverage of multiple detection systems strategically deployed on the infrastructure under a common distributed framework, which permanently correlates all detected malicious patterns and anomalies and learns from them.

The study and modeling of opinion propagation in a network through the interactions of its agents originated a few decades ago. French in 1956 was one of the first researchers who focused on opinion dynamics [34]. Subsequently, De Groot formalized one of the simplest and most famous dynamic models of opinions in 1974 [35]. Since then, the interest of the research community has gradually increased and according to the nature of the context under consideration the format of the different opinions expressed by the agent and the purpose of interaction, the dynamics of opinions have taken different forms [36,37,38].

Opinion Dynamics can be used in the SG cybersecurity context to design a multi-agent advanced detection system [30,31,32,39], which is one of the main defense threats in the field of SG cybersecurity [40]. In [28], an intrusion detection scheme Opinion Dynamics-based was initially proposed under a theoretical perspective. From a practical point of view, in [31] its ability to detect and monitor attacks in an industrial testbed was demonstrated; in [40], it also showed its contributions to the Smart Grid scenario, and in [32] to the Industrial IoT, also known as IIoT, scenario. This is possible because the opinion dynamics can include anomalous indicators (i.e., equipment and communication link compromises) as the main indicators (opinions), which also include the integration of external IDS.

## 4. SAMGRID Concept

**Figure 2:**
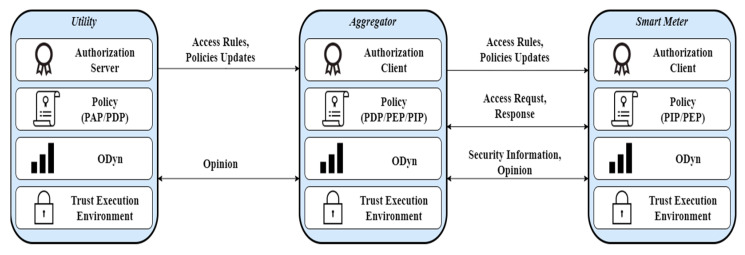
SAMGRID architectural components.

In this section, we present an archetype of the proposed module along with the processes that take place for their consistent integration as shown in Figure 2 with any SG application. In SAMGRID, apart from the standard entities and roles that participate in SG authorization process (see Section 2) we introduce an opinion dynamics (ODyn) mechanism (see Table 2), an additional mechanism that we consider as the pedestal of our module. *ODyn* is a mechanism that in a continuous basis *monitors* a specific domain of the grid, enforcing the participating entities to exchange security information among them. *ODyn* is also utilized to guarantee the integrity and availability of each active entity. We have to note, that the proposed authorization process as well as the *ODyn* utilize TEE [41] that is the anchor for integrity and validation proofs constructing a robust foundation for any application to be on top; however, we will avoid analyzing the TEE and its performance because TEE is out of scope of this work.

### 4.1. SAMGRID

SAMGRID consists of two individual sub-modules. The first one is the *Authorization* that handles issues related to issuing and enforcing policies within a SG domain. The second sub-module is *ODyn*, which is responsible for monitoring the security status of each participating node, understanding if a node is compromised or not. 

In the case where a new node is introduced into a SG domain, the first executed process will be part of the *Authorization* sub-module so that it can get updated regarding the latest policies (e.g., security policies). Once the processes of the *Authorization* submodule are successfully completed, the *ODyn* then starts working since the new node shall start exchanging the respective information. The same pattern is followed also when the *Authorization* sub-module will issue and demand the spread of a brand-new policy. To perform the SAMGRID operations, we assume that SMs, Aggregators and Utilities are the participating parties and have at least one of the available roles (PAP, PDP, PEP and PIP) (see also Table 2). For instance, a Utility may be at the same time a PAP and a PDP.

### 4.2. SAMGRID Authorization

First, we need to properly define the mechanism behind the Authorization module. As established previously the Authorization module is the one responsible (and that holds the authority) to accept or deny requests within the SG, be that a reallocation of resources or the integration of a new device. What this essentially means is that the state of the SG is continuously changing due to its nature (varying loads within the grid for example), and it is readjusting itself to be as optimal as possible. Therefore, we have a set of factors that change the state of the SG due to the needs that it serves, and we have a set of local components that try to change the state of the SG in order to properly adapt it. The actions of the latter need to be properly analyzed before they are accepted in order to ensure that the new SG state that they will create, will not be a vulnerable one or even become damaged either intentionally (malicious intent) or unintentionally (accidental). In order to accomplish that SAMGRID proposes a hierarchical authorization framework, composed of the previously defined roles (PAP, PDP, PIP, PEP). 

In order to know the new state of the SG that accepting a request will create previous states must be known. This is conducted using entities that hold the PIP role. These are located essentially everywhere in the Smart Grid and their role is to gather as much information as possible. This is conducted using the embedded context-awareness module, present in all devices within the grid. The data gathered are normalized in order to keep only relevant information, the rest being dismissed as noise. This information can consist for example of the number of energy usage readings, the set of households controlled by an aggregator and their energy demands, updates regarding utility systems, databases, contractual arrangements, and network related aspects. Security assessments are also being conducted at this level in order to enable a fast response in the case of a potential threat. This is mainly accomplished by the Opinion Dynamics module that will be discussed in the next section. 

With the current state, which is essentially a digital twin, of the Smart Grid known, the next state created by a request can be predicted. In order to assess if the new state is desirable (does not contain security threats, for example), strict policies are defined. The entities that tackle the control-access policies are the ones that have a PDP role.

### 4.3. SAMGRID Opinion Dynamics (ODyn)

In this section, we will analyze *ODyn*’s goals and technical approach. *ODyn* relies on numerous internal processes and only their combination can lead to its final target.

SAMGRID adopts and integrates an opinion dynamics approach that aims to seamlessly work together with the SAMGRID authorization mechanism intending to address and maintain a secure ecosystem with a low cybersecurity risk. In particular, *ODyn* transforms the nodes, which participate in a domain, from being passive without being involved with security actions, to active agents. The latter, due to the new activated mode, are enforced to communicate among them security related information to detect anomalies. As previously clarified (see Section 2), PDP, a role mainly given to a Utility, is capable to authorize access to the grid resources based on policies or on the security status of the controlled domain as well as various attributes (e.g., usage of computational resources) that affect the assets that request access. The latter information is provided at all times by the *ODyn* module that is hardcoded in each participating node of a domain. Overall, *ODyn* acts as a framework that gathers and combines input from various sources (due to heterogeneity of a domain). The combination is crucial for monitoring a domain and confronting security threats in it, during its whole wheel of life. *ODyn* is capable of it through its correlation algorithm that analyzes and traces numerous threats. 

In particular, our module, *ODyn*, follows pre-existing models [28,31] perceives a domain as a graph *G*(*V*,*E*), where *V* is the set of nodes {$v_{i},\ldots ,v_{n}$} and *E* is the set of edges {$e_{i},\ldots ,e_{m}$} that represent the connection ($v_{i},v_{j}$) among the nodes. Moreover, there is the set *A = {*$a_{1},\ldots ,a_{n}$*}* that mirrors the agents. As we described above, each node due to the *ODyn* has been transformed to an agent; this leads to fact that |*V*|=|*A*|. ODyn aims to compute the opinion of an agent $a_{i}$ in the tth iteration, we defined it as $o_{i}\left(t\right)$. It receives values from 0 to 1, where 0 means the absence of anomaly while 1 shows the paramount anomaly. To compute this value, every agent $a_{i}$ nominates a value—its opinion—to its neighbor j to consider or not its opinion that is denoted as $w_{ij}$. We have to note that the sum of weights coming from every agent is 1, regarding its own opinion. Based on the aforementioned assumptions, *ODyn* calculates the opinion of agent $a_{i}$ in the iteration t+1 based on the following function $o_{i}\left(t+1\right)=w_{i1}o\left(t\right)+w_{i2}o\left(t\right)+\ldots +w_{in}o\left(t\right)$. We can observe that the influence on a specific agent comes from a weighted average of the opinions that stem from its neighbors.

## 5. Performance Evaluation

In this section, we analyze the performance evaluation of the submodules (see Section 4) that assemble the SAMGRID component to scrutinize the feasibility and efficiency of the proposed module. 

### 5.1. Authorization

We analyzed the performance of the Authorization component of SAMGRID in order to gather insight on its feasibility and scalability. We have tested the response time of the authorization API (as a crucial parameter that might otherwise render the component unusable) as well as the RAM and CPU consumption. In our proof-of-concept implementation, the participating SMs and Aggregator are simulated on ARM devices and used as operating system Raspbian Jessie. The Utility, which is the owner of the domain, is a server with Intel(R) Core (TM) i5-65000 processor. The testbed is summarized in Table 3.

To assess the authorization component, we subjected it to a stress test, using a series of scripts written in bash, as well as two tools: *percentile* (https://github.com/yuya-takeyama/percentile (accessed on 17 March 2022)) and *ntimes* (https://github.com/yuya-takeyama/ntimes (accessed on 17 March 2022)).

We defined a series of tests by varying the number of clients that call the REST API interface of the component. We considered as evaluation results the measured API response time as well as CPU and RAM variation according to the number of clients.

Table 4 shows the performance of the authorization component as deployed on the three types of the SAMGRID devices. We can observe from the results that the CPU utilization is relatively low with a few percentages for all device types, especially for a small number of clients making requests. We noticed an increase in CPU utilization especially for the Aggregator and Utility. The memory consumption of the SAMGRID devices does not vary significantly with the number of connected clients. For the SM and Aggregator, it is relatively constant at approx. 30% of RAM consumed. For the utility, it drops to 18–25% of RAM.

The most significant difference is noticed in API response time. The more powerful resources of the Utility enable a faster response time, ranging from 38 ms to 1.54 s. For the SM, these numbers are considerably worse (starting from 162 ms to 6.6 s), as well as for the Aggregator (from 71 ms to 2.5 s).

### 5.2. ODyn

Regarding the *ODyn*, we analyzed the performance of this sub-module of SAMGRID to investigate its feasibility and effectiveness. We focused on the execution time of the core source code of *ODyn* regarding the time needed to detect a cyber-attack within a specific domain, identify the malicious node as well as to spread the opinion of the latter node to the whole domain. For our proof-of-concept implementation, the participating SMs and Aggregator bearing the responsibility of executing the *ODyn* are simulated on ARM devices with a 700 MHz single-core CPU, a 512 MB RAM and used as operating system Raspbian Jessie. The Utility, which is the owner of the domain, is a server with Intel(R) Core (TM) i5-65000 at 3.2 GHz with four cores, an 8GB RAM and used as the operating system Debian GNU/Linux 10. The testbed is summarized in Table 5.

For the *ODyn* prototype, we developed and utilized our own implementation of *ODyn* in Python language and forced one iteration (see Section 4) per second; it is a custom implementation and can be configured to match the various cases. Moreover, to simulate the ransomware and crypto mining attacks, we used our own scripts in Pythons to deplete the sources of the infected nodes; the latter were randomly chosen by a Python script and the *malicious code* was then integrated to their OS.

To assess the performance of *ODyn*, we calculated the average CPU utilization, memory consumption and network usage during every iteration of the *ODyn* algorithm as shown in Table 6. We evaluated the behavior of *ODyn* in three different cases where the domain had a different length including 100, 500 and 1000 SMs and Aggregators. To calculate the aforementioned values, we executed the experiments 10 times. We have to note that we do not consider the Utility as an extra domain participant since it participates in every experiment as the owner. Regarding the CPU consumption of the participating SMs and Aggregator it is constant at 1.44% regardless of the domain length. However, the CPU utilization for the Utility, which is the owner of the domain, fluctuated based on the domain length. As it is presented in Table 4, when the domain included 100 nodes, then the CPU utilization was at 1.47%, with 500 nodes was 18.07%, while when within the domain, there were 1000 nodes the CPU utilization reached the 25% of the CPU (one core out of four available cores). Furthermore, the memory consumption for the participating nodes of the domain was consistent disregarding the length of the domain. In addition, the memory consumption at the Utility’s side is at 0.26%, 0.9% and 1.1% for a domain including 100, 500 and 1000 nodes, respectively. Additionally, the network usage of SMs and Aggregator regards their number was steady at 514 Kb per node. While the network usage at the Utility’s side escalated based on the domain’s length. In particular, it was 51 MB, 255 MB and 500 MB for a domain including 100, 500 and 1000 nodes correspondingly. Overall, based on our experiments, we can observe that the *ODyn* as an individual component of SAMGRID does not deplete the resources of the participating entities, even for constrained devices such as SMs and Aggregators. Additionally, based on the results we can assume that the *ODyn* does not impede the rest of the SG application that may work on top of the SAMGRID enabling their seamless cooperation.

**Figure 3:**
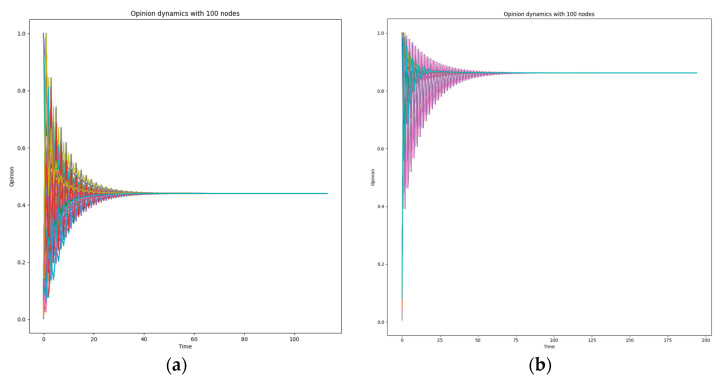
(**a**) 10 out of 100 nodes are infected. (**b**) 55 out of 100 nodes are infected.

Finally, we assessed the effectiveness of the *ODyn* algorithm against ransomware and crypto mining attacks, developing Python scripts, to investigate if our implementation can identify the attack and detect the malicious node in time. To complete the aforementioned experiment, we created a domain with one Utility that is the owner of the domain, and 100 nodes from SMs and Aggregators. In both simulated cyber-attacks (ransomware and crypto mining), we randomly infected 10 and 55 nodes to examine how responsive the *ODyn* is. We have to note that, in this evaluation, to calculate the corresponding values, we executed the experiments 10 times, as we did for the previous experiments (see previous paragraph). In particular, we targeted to scrutinize if the final opinion that is generated, for a specific node due to a cyber-attack, by the algorithm (see also Section 4) is unbiased or not by its neighbors. We have to note that the graph used in both cyber-attacks is a fully connected graph. We decided to follow this approach to provoke *ODyn* to follow a false opinion; it could lead to a silent cyber-attack with numerous aftermaths (see Section 2). On the one hand, during the first experiment, the 10 out 100 nodes are detected as infected nodes since their opinion starts with value being from 0.8 to 1, the rest nodes are healthy having low values as opinions. In Figure 3a, we can observe that in the first iterations (Time—*x* axis) the opinion is different and quickly we understand that the domain is under attack. However, while the iterations pass the opinion dramatically changes creating the opinion that the domain is healthy and not under attack, the opinion was near to 0.5 (see Figure 3a). On the other hand, when the infected nodes were 55 out of the 100 nodes the situation was totally different. In the first iterations the opinion was steady high. However, this opinion was followed even after the 100 iterations. Then, the total opinion was near to 0.9 suggesting that the domain is under attack (see Figure 3b). Overall, studying the results we can observe that the final opinion of a domain depends on the connectivity among the participating nodes, the number of the infected nodes and the approach being encapsulated by each Utility regarding the value of each opinion (extreme; high; medium; low). It is discernible that the more the connections among the nodes, the more accurate the opinion. However, the most important is that *ODyn* can detect that a domain is under attack when the number of infected devices is more than half of the total participating. Finally, we have to note the final opinion in the question “Is the domain under attack or not?” depends on the approach that is followed by each Utility against the *ODyn*. The stricter the used approach is the more agile the opinion will change.

## 6. Security Analysis

In this section, we evaluate the security level provided by SAMGRID in relation with the security requirements presented in Section 2. We argue that SAMGRID meets all security requirements presented in Section 2, except for physical protection (S5 requirement as presented in Section 2), as hardware security [42] can be considered out of the scope of this work. First, the SAMGRID integrating the Authorization sub-module achieves maintenance within a domain specific entity that follow the security policies being issued by their corresponding PDPs and PAPs. Apart from this, SAMGRID, having numerous nodes with the PIP in a network topology, can gather data and through the *ODyn* can evaluate the behavior of each participating node based on specific policies. We can observe that the collaboration of the two SAMGRID sub-modules is capable of safeguarding the data confidentiality in a domain.

Moreover, the Authorization sub-module that is based on FI-WARE (see Section 4) is responsible for monitoring the various events, issue policies (e.g., security policies) and audit the actions of the participating nodes within a domain. In particular, our implementation utilizes cloud as we have already clarified in Section 4 and all events and logs are stored there. Through the audit of the various events, SAMGRID is based on the issued security policies and identifies malicious events and actors who tried to violate any policy. Thus, SAMGRID can practically maintain the accountability and non-repudiation features that are crucial in a SG domain. Additionally, *ODyn* utilizes TEE for its main processes. Its integration effectively enriches the integrity and authenticity features. TEE, due to its characteristics, safeguards against alteration and replication attacks. However, we do not analyze here the TEE since we have analyzed its capabilities and performance in our previous published work [41]. 

Finally, a hypothesis indirectly related to the security characteristics of the SAMGRID is that instead of designing new protocols from scratch, we have appointed a solution that includes a long-established technology. More specifically, SAMGRID’s pillars are technologies that have been broadly analyzed and reviewed, and up to now, there are no imminent threats that can break its security properties. This makes SAMGRID not only provably secure, but also easier to be absorbed by industrial environments.

## 7. Conclusions

This work introduces, for the first time, the SAMGRID module that combines an authorization mechanism and an opinion dynamics approach (*ODyn*) to maintain and spread a standard cybersecurity risk level in a SG domain. The security status of a SG domain depends not only on the security risk inherited by the components but also on the inadequate security controls implemented by handlers to these. The crux of the SAMGRID is the *ODyn* that monitors on a continuous basis the security status of all participating components of a SG domain. Having designed and implemented the SAMGRID, we quantitatively evaluated its performance and effectiveness. One the one hand, regarding its performance we proved that it could cope with various domains regardless of their length (continuous join and leave actions of components). On the other hand, we assess SAMGRID and especially *ODyn*’s effectiveness against attacks that simulated ransomware and crypto mining attacks. We believe that this work will pave the way for numerous upcoming schemes and frameworks for enhancing the security features of the SG ecosystem, as we did with the recent introduction of SAMGRID.

The outcomes of this paper can be extended in various ways as a future work. For this proof-of-concept implementation of SAMGRID, we designed and developed a prototype; *ODyn* was evaluated in simulation environments, while the authorization module was evaluated in a virtualized environment. Next, we plan to integrate SAMGRID as a whole in physical devices assessing its behavior. Moreover, we aim to utilize self-sovereign-identity technologies to assess SAMGRID’s applicability in the SG ecosystem. This will help us to identify supplementary case studies for SAMGRID to advance its current features and extend its functionalities with new ones.

## Figures and Tables

**Table 1:** SealedGRID features. ✓shows the enabled features of the system.

Related Work/Features	Blockchain	Access Control	Opinion Dynamics	Machine Learning	Data Integrity	Interoperability
Smart Grid Interoperability Standards [4]						✓
Interconnection between heterogeneous cyber-physical systems [5]						✓
Blockchain technology for smart grids [6]	✓				✓	
ML models for Electricity Theft Detection [7]				✓	✓	
Load frequency control of smart grids [8]						
Confidentiality in Smart Grids [9]				✓	✓	
Metering and data access infrastructures in smart grid [10]		✓			✓	
Policy enforcement in smart grid [11]		✓				
Access control for smart grid services based on publish/subscribe [12]		✓				✓
Blockchain-based authorization system [13]	✓				✓	
Proposed work	✓	✓	✓	✓	✓	✓

**Table 2:** Main entities and roles participating in SAMGRID.

Entity	Description
Smart Meter (SM)	Collect the readings of the electricity consumption.
Aggregator	Sum all the SMs’ readings and transmit the result to the Utility.
Utility	Compute the total consumption of a customer.
**Role**	**Description**
PAP	Authors and maintains a set of policies.
PDP	Assesses a policy against other relevant policies and attributes.
PIP	Is a source of attribute values.
PEP	Performs decision requests, receives policy updates and accordingly translates them, and enforces policies’ decisions.

**Table 3:** SAMGRID testbed parameters for Authorization.

Entity	Setup
Smart Meter	- ARM Device, 4-core CPU at 1–1.2 GHz, 512 MB RAM
Aggregator	- ARM Device, 4-core CPU at 1.2–1.4 GHz, 1 GB RAM
Utility	- Intel Core i5-6500 CPU at 2 GHz 8 cores, 8 GB RAM

**Table 4:** Authorization component performance.

# of Nodes	Average CPU Utilization (Percentage)			Average Memory Consumption (MB)			API Response Time (ms)		
	Smart Meter	Aggregator	Utility	Smart Meter	Aggregator	Utility	Smart Meter	Aggregator	Utility
10	6.86	1.39	0.8	154.57	315.69	1455.42	162.85	71	38.62
50	6.68	6.87	3.43	154.71	327.29	1464.28	868.83	357.41	137.96
100	6.58	12.82	6.59	132.28	261.78	1481.81	4041.98	460.3	189.1
500	7.86	19.3	34.3	137.40	304.12	2033.95	6670.35	2529	1545.4

**Table 5:** SAMGRID testbed parameters for ODyn.

Entity	Setup
Smart Meter, Aggregator	-ARM Device single-core CPU at 700 MHz, 512 MB RAM (Download: 9.6 Mbps; Upload: 9 Mbps)
Utility	-Intel Core i5-6500 CPU at 3.2 GHz 4 cores, 8 GB RAM (Download: 98 Mbps; Upload: 92 Mbps)

**Table 6:** ODyn overhead.

# of Nodes	CPU Utilization (Percentage)		Memory Consumption (Percentage)		Network Usage	
	Smart Meter, Aggregator	Utility	Smart Meter, Aggregator	Utility	Smart Meter, Aggregator	Utility
100		1.47		0.26		51 MB
500		18.07		0.9		255 MB
1000	1.44	25	0.1	1.1	514 Kb	500 MB

## Data Availability

Not applicable.

## References

[1] Khan M.W., Wang J., Ma M., Xiong L., Li P., Wu F. (2019). Optimal energy management and control aspects of distributed microgrid using multi-agent systems. Sustain. Cities Soc..

[2] Ghosal A., Conti M. (2019). Key management systems for smart grid advanced metering infrastructure: A survey. IEEE Commun. Surv. Tutor..

[3] Rendroyoko I., Setiawan A.D. (2021). Development of Meter Data Management System Based-on Event-Driven Streaming Architecture for IoT-based AMI Implementation. Proceedings of the 2021 3rd International Conference on High Voltage Engineering and Power Systems (ICHVEPS).

[4] Gopstein A., Nguyen C., O’Fallon C., Hastings N., Wollman D. (2021). NIST Framework and Roadmap for Smart Grid Interoperability Standards, Release 4.0.

[5] Alcaraz C., Lopez J. (2020). Secure interoperability in cyber-physical systems. Cyber Warfare and Terrorism: Concepts, Methodologies, Tools, and Applications.

[6] Hasankhani A., Hakimi S.M., Bisheh-Niasar M., Shafie-khah M., Asadolahi H. (2021). Blockchain technology in the future smart grids: A comprehensive review and frameworks. Int. J. Electr. Power Energy Syst..

[7] Javaid N., Gul H., Baig S., Shehzad F., Xia C., Guan L., Sultana T. (2021). Using GANCNN and ERNET for Detection of Non-Technical Losses to Secure Smart Grids. IEEE Access.

[8] Yang H., Liu S., Fang C. (2020). Model-based secure load frequency control of smart grids against data integrity attack. IEEE Access.

[9] Boudko S., Aursand P., Abie H. (2020). Evolutionary Game for Confidentiality in IoT-enabled Smart Grids. Information.

[10] Veichtlbauer A., Engel D., Knirsch F., Langthaler O., Moser F. Advanced metering and data access infrastructures in smart grid environments. Proceedings of the Seventh International Conference on Sensor Technologies and Applications (SENSORCOMM) 2013.

[11] Alcaraz C., Lopez J., Wolthusen S. (2016). Policy enforcement system for secure interoperable control in distributed smart grid systems. J. Netw. Comput. Appl..

[12] Duan L., Liu D., Zhang Y., Chen S., Liu R.P., Cheng B., Chen J. (2016). Secure data-centric access control for smart grid services based on publish/subscribe systems. ACM Trans. Internet Technol. (TOIT).

[13] Alcarria R., Bordel B., Robles T., Martín D., Manso-Callejo M.Á. (2018). A blockchain-based authorization system for trustworthy resource monitoring and trading in smart communities. Sensors.

[14] Bolgouras V., Ntantogian C., Panaousis E., Xenakis C. (2019). Distributed key management in microgrids. IEEE Trans. Ind. Inform..

[15] Karopoulos G., Ntantogian C., Xenakis C. (2018). MASKER: Masking for privacy-preserving aggregation in the smart grid ecosystem. Comput. Secur..

[16] Farao A., Veroni E., Ntantogian C., Xenakis C. (2021). P4G2Go: A Privacy-Preserving Scheme for Roaming Energy Consumers of the Smart Grid-to-Go. Sensors.

[17] Suciu G., Istrate C.I., Vulpe A., Sachian M.A., Vochin M., Farao A., Xenakis C. Attribute-based access control for secure and resilient smart grids. Proceedings of the 6th International Symposium for ICS & SCADA Cyber Security Research 2019.

[18] Suciu G., Istrate C., Sachian M.A., Vulpe A., Vochin M., Farao A., Xenakis C. FI-WARE authorization in a Smart Grid scenario. Proceedings of the 2020 Global Internet of Things Summit (GIoTS).

[19] Langner R. (2011). Stuxnet: Dissecting a cyberwarfare weapon. IEEE Secur. Priv..

[20] FireEye (2015). Cyber Attacks on the Ukrainian Grid: What You Should Know. https://www.fireeye.com/content/dam/fireeye-www/global/en/solutions/pdfs/fe-cyber-attacks-ukrainian-grid.pdf.

[21] Kaspersky BlackEnergy APT Attack in Ukraine. https://www.kaspersky.com/resource-center/threats/blackenergy.

[22] Wired The Untold Story of a Cyberattack, a Hospital and a Dying Woman. https://www.wired.co.uk/article/ransomware-hospital-death-germany.

[23] Nbc News Colonial Announces Pipeline Restart, Says Normal Service Will Take ‘Several Days’. https://www.nbcnews.com/tech/security/colonial-announces-pipeline-restart-says-normal-service-will-take-seve-rcna917.

[24] Hackers Breached Colonial Pipeline Using Compromised Password. https://www.bloomberg.com/news/articles/2021-06-04/hackers-breached-colonial-pipeline-using-compromised-password.

[25] Farao A., Panda S., Menesidou S.A., Veliou E., Episkopos N., Kalatzantonakis G., Mohammadi F., Georgopoulos N., Sirivianos M., Salamanos N. (2020). SECONDO: A platform for cybersecurity investments and cyber insurance decisions. Proceedings of the International Conference on Trust and Privacy in Digital Business.

[26] The Cyber Kill Chain. https://www.lockheedmartin.com/en-us/capabilities/cyber/cyber-kill-chain.html.

[27] Yadav T., Rao A.M. (2015). Technical aspects of cyber kill chain. Proceedings of the International Symposium on Security in Computing and Communication.

[28] Rubio J.E., Alcaraz C., Lopez J. Preventing advanced persistent threats in complex control networks. Proceedings of the European Symposium on Research in Computer Security.

[29] Mitre Att&Ck. https://attack.mitre.org/.

[30] Nisioti A., Loukas G., Laszka A., Panaousis E. (2021). Data-driven decision support for optimizing cyber forensic investigations. IEEE Trans. Inf. Forensics Secur..

[31] Rubio J.E., Roman R., Alcaraz C., Zhang Y. (2019). Tracking apts in industrial ecosystems: A proof of concept. J. Comput. Secur..

[32] Rubio J.E., Roman R., Lopez J. (2020). Integration of a threat traceability solution in the industrial internet of things. IEEE Trans. Ind. Inform..

[33] “OpenWay Riva”, Itron. https://blogs.itron.com/tag/openway-riva/.

[34] French J.R. (1956). A formal theory of social power. Psychol. Rev..

[35] DeGroot M.H. (1974). Reaching a consensus. J. Am. Stat. Assoc..

[36] Dong Y., Zhan M., Kou G., Ding Z., Liang H. (2018). A survey on the fusion process in opinion dynamics. Inf. Fusion.

[37] Noorazar H. (2020). Recent advances in opinion propagation dynamics: A 2020 survey. Eur. Phys. J. Plus.

[38] Grabisch M., Rusinowska A. (2020). A Survey on Nonstrategic Models of Opinion Dynamics. Games.

[39] Lopez J., Rubio J.E., Alcaraz C. (2018). A resilient architecture for the smart grid. IEEE Trans. Ind. Inform..

[40] Gunduz M.Z., Das R. (2020). Cyber-security on smart grid: Threats and potential solutions. Comput. Netw..

[41] Suciu G., Sachian M.-A., Vulpe A., Vochin M., Farao A., Koutroumpouchos N., Xenakis C. (2021). SealedGRID: Secure and Interoperable Platform for Smart GRID Applications. Sensors.

[42] Muñoz A., Farao A., Correia J.R.C., Xenakis C. (2021). P2ISE: Preserving Project Integrity in CI/CD Based on Secure Elements. Information.

